# Author's reply

**Published:** 2009

**Authors:** Peter Diel, Dominique Merky, Christoph Röder, Albrecht Popp, Malgorzata Perler, Paul Ferdinand Heini

**Affiliations:** 1 *Spine Service, Inselspital Bern, Bern, Switzerland*; 2 *Osteoporosis Policlinic, University Hospital, University of Bern, Bern, Switzerland*; 3 *Institute for Evaluative Research in Orthopedic Surgery, University of Bern, Bern, Switzerland*

Sir,

We are thankful for this comment^1^ and agree with the colleague's opinion. The usefulness of vertebroplasty^2^ should not only be evaluated against the conservative pharmacological treatment but also against newer technologies, like balloon kyphoplasty or vertebral body stenting. The cost-effectiveness study, which is a methodologically challenging study on its own, must include not only pain alleviation but also reoperation caused by new vertebral fractures. We believe that vertebroplasty is, currently, the only technique suitable for prophylactic augmentation and that this approach further increases its cost-effectiveness. As for the availability, the procedure can be performed with very basic equipment: a special formula of polymethylmethacrylate (PMMA) with a high radioopacity is one prerequisite for a safe procedure. Then, one can use simple disposable 8- or 10-gauge biopsy cannulas and inject the cement with 2 and 1 cc syringes. In order to control the cement flow only high-viscosity cement should be injected. A three-level injection accounts for about 250 euros. This is the way it was developed and performed in the earlier days with convincing outcomes and it can still be applied like that if no other material resources are available.
